# "The Hospital" Nursing Mirror

**Published:** 1897-04-10

**Authors:** 


"The Hospital" Nursing Mirror.

Being the Nursing Section of "The Hospital."

[Contributions for this Section of "The Hospital" should be addressed to the Editor, The Hospital, 28 & 29, Southampton Street, Strand, London, W.C., and should have the word "Nursing" plainly written in left-hand top corner of the envelope.]

### News from the Nursing World.

### THE QUEEN'S COMMEMORATION.

The fund which is being raised in commemoration of the Queen's reign on behalf of the Queen Victoria Jubilee Institute for Nurses has now amounted to £48,971. The committee are making every effort to increase this sum to at least £100,000 before June 22nd.

### A LOSS TO WINDSOR.

The committee of H.R.H. Princess Christian's "Trained Nurses" at Windsor have received with great regret the resignation of Miss Simpson, who has been lady superintendent for the past five and a half years, and who is now giving up nursing to enter a sisterhood. Miss Simpson's loss will be greatly felt at Windsor, where she will be missed alike by the Princess and the members of the committee, by the nurses, and by the poor. At the recent annual meeting the following resolution was proposed by the Dean of Windsor, seconded by the Mayor, and carried unanimously: "That this committee deeply regrets the loss which the Home will sustain by the loss of Miss Simpson, the lady superintendent, and desires to place on record its thankful appreciation of the great services rendered to the Home during the anxious years of its early growth and development by Miss Simpson's capacity and tact and thorough knowledge of all the requisite details of the work, which have contributed so largely towards the position of increasing usefulness which the Home has now reached." The committee have appointed Miss Robin, superintendent of the Nurses' Home, Edinburgh Royal Infirmary, to succeed Miss Simpson.

### QUEEN'S NURSE AT REDDITCH.

The annual meeting of the Redditch District Nursing Association was held on March 31st, presided over by Mr. J. F. Milward, J.P., and was well attended, in spite of the bad weather. Canon Newton (president and chairman of committee) was present. The ninth annual report was passed and approved, and the services of the hon. secretary (Mrs. Page) gratefully acknowledged. After the transaction of the formal business of the meeting, Miss Gethen (formerly Sister Queen Victoria, of the London Hospital) gave an interesting and much appreciated address on "Nursing, Past and Present," dwelling specially on the undoubted economy of providing trained and competent district nurses for the sick poor in their homes. A presentation was afterwards made to Nurse Deville (Queen's nurse), who has given great satisfaction to the committee and to her patients for nearly eight years. It consisted of £25, which was handed to the nurse in a handsome green morocco purse, accompanied by an embroidered satin case (the work of Miss Milward) containing the words, "Presented to Nurse Deville by the members of the Nursing Committee and other friends, who wish to show their appreciation of her good work amongst the poor of the town." The report shows that 7,451 visits were paid last year to 268 cases, 240 being of an acute, and 28 of a chronic, nature. Nurse Devile has received valuable assistance for some time past from Miss Shore, a resident of Redditch, but it is hoped that, at some future day, funds may be forthcoming for the maintenance of a second Queen's nurse, the services of the one already installed having been so cordially appreciated.

### ENGLISH NURSES FOR CRETE.

The following announcement appeared in *The Globe* for April 7th: "To-morrow morning a special saloon carriage will convey the following seven ladies, who will be dressed in their uniform as nurses, from Charing Cross to Dover, *en route* to Athens for Crete—Lavinia Foukes, Charlotte Flanagan, Beatrix Farnsworth, Lillie Warriner, Lilian Lees, Emma Curtise, and Mrs. Ormiston Chant. The Dover people will give the lady volunteers a send-off. Madame Parrans and ladies of the Red Cross Society, who will join the English lady volunteers, go direct to Crete. The Foreign Office have, through the Admiralty, given instructions that every facility should be given to the English nurses on their arrival by the Admirals of the English fleet." We need not say that we wish these ladies a pleasant trip.

### THE MALE NURSES' (TEMPERANCE) CO-OPERATION.

The Male Nurses' (Temperance) Co-operation reports that the third year of its existence has been a most successful one. The staff has increased, and numbers nearly 100 members, who find constant employment, the average earnings for each man for the year working out at the rate of £101 18s. 2d., after paying all expenses. The Co-operation is under the patronage of Sir James Crichton Browne, Dr. Lauder Brunton, Professor Victor Horsley, and other well-known medical men. The rules state that only men who have had not less than three years' training, and possessing at least two medical references in addition to certificates of personal character, are registered on the books of the society, while all masseurs sent out by it must hold a certificate from the National Hospital. A Benefit Society has lately been started for the advantage of members during illness.

### WICK AND PULTENEY TOWN DISTRICT NURSING ASSOCIATION.

The Wick District Nursing Association (affiliated to the Q.V.J.I.) is lamenting the loss of its energetic secretary, Mrs. Gunn, who is leaving Wick to live in London. Mrs. Gunn was the originator of the district nursing scheme in 1893, and in her capacity of hon. secretary has devoted herself to its success ever since. Mrs. Leith has been appointed to succeed her as hon. secretary to the association.

### NURSES' UNIFORMS.

At a meeting of a Nurses' Debating Society the other day some interesting speeches were made on the question "Ought we to get up a Petition praying for the Protection of our Uniforms?" A show of hands at the conclusion of the discussion resulted in a majority of 42 against 3 that "some action must speedily be taken to suppress the wearing of uniform by any but accredited hospital nurses."

### A LADY FACTORY INSPECTOR FOR NEW SOUTH WALES.

MISS ANNIE DUNCAN, who has just been appointed to the office of Inspector of Factories in New South Wales, is the first woman to hold such a post in the Colony. Miss Duncan has done good work in this country as sanitary inspector under the Kensington Vestry, and she is an Associate of the Sanitary Institute and of the National Health Society. She is an Australian by birth.

### NURSING IN INDIA.

A SUGGESTION was recently made in the *Times of India* to organise in Bombay a fund for providing trained nurses in the local hospitals. Another Indian paper follows it up by proposing that such a fund might be started at the present time in commemoration of Mr. Birdwood, a retiring member of the council, who has "universally endeared himself to the entire population of this city and the Presidency." The writer adds that "no movement to commemorate his services in India would . . . be more appropriate than to establish a fund in his honour for providing trained nurses for the numerous hospitals of this city, which are sadly in need of their humane services."

### QUEEN'S COMMEMORATION AT BRIGHTON.

THE Countess of Chichester presided over a well-attended meeting held at Brighton to consider the question of the Queen's Commemoration, and made an eloquent appeal on behalf of the Brighton Branch of the Queen's Jubilee Institute, to endow which would, she said, be not only for the lasting good of the poor of the town, but a jubilee effort which would be acceptable to the Queen. Speeches were made by Mrs. Wilberforce, Mrs. Scharlieb, Miss Buckle, and others, and a resolution was passed that "in commemoration of the Diamond Jubilee of her Most Gracious Majesty, the women of Brighton shall raise a fund to endow the local branch of the Queen Victoria Jubilee Institute for Nurses."

### COUNTESS OF DUFFERIN'S FUND.

A VERY encouraging report was presented at the annual meeting of the Countess of Dufferin Fund for Supplying Medical Aid to Women in India. A great work has yet to be done, but there is most satisfactory evidence that rulers of native States and native associations are now lending their aid to the furtherance of the scheme. Lord Elgin, who presided at the meeting, in speaking of the famine and the suffering it has brought in its train, said that he had received many letters written by the Queen herself, expressing her great sympathy with her Indian subjects in the season of misery and disease through which they are passing.

### THE ZENANA MISSION IN INDIA.

MR. STOKES, an American gentleman, has given a donation of Rs. 1,307 to the Lady Kinnaird Memorial Hospital at Lucknow, for the purpose of defraying the cost of one of the smaller wards in the new wing, opened at the beginning of this year. The ward will be called the "Stokes Memorial Ward," and is named in memory of Mr. Stokes' wife and daughter. The Hon. Gertrude Kinnaird is undertaking a visit to the Riviera this spring, with the object of spreading information about the work of the mission and collecting funds.

### AN EXCELLENT ARRANGEMENT.

MISS NOTT BOWER has started a plan for the comfort of her night nurses, which it must be hoped will soon be the rule and not the exception in all hospitals. The night staff at Guy's has been increased of late, and this addition has made it possible for every nurse to have a clear half-hour away from her ward during the night to get a good and substantial meal. This is laid in the Home, and consists of cold joints, cheese, jam, bread and butter, &c. The nurses thoroughly appreciate this new arrangement, which enables them to have a really comfortable meal, and sends them back to their wards rested and refreshed. Of course, they can make their own tea, &c., while on duty as usual; but the chief meal of the night can now be enjoyed at leisure, and right away from the atmosphere of the wards.

### ROYAL NATIONAL PENSION FUND FOR NURSES.

THE secretary of the Pension Fund has asked us to find space in our columns to thank the very numerous policyholders who have written kind letters of appreciation of the way the Fund has been managed for them, which have come to the office since the issue of the annual report. We are very pleased to do this, as nurses will be gratified to know that their kindly thought in writing gives genuine pleasure, and is a reward for very arduous and zealous services so freely, as they all know, rendered on their behalf.

### KILMARNOCK NURSING ASSOCIATION.

THE Kilmarnock Nursing Association reports a busy year. Three nurses have been at work, assisted by temporary help from February to August, and there was a considerable increase in the number of cases visited, and in operation work. The committee remark that they have observed with much satisfaction "that this increase of work and responsibility was cheerfully undertaken by the nurses, and desire here to publicly acknowledge the same." Miss Finnie, of Springhill, has been appointed vice-president in place of Mrs. Dunlop, of Annanhil, who has resigned. The association is able to show a balance of income, from subscriptions and donations, over expenditure of £17 0s. 11d.

### SHORT ITEMS.

THE Queen has accepted a presentation copy of "Behind the Great Wall," a story of the work of the Church of England Zenana Mission in the Fuh-Kien province of China.—*Black and White* for April 3rd has a photograph of Nurse Ellen Joyce, who died recently at Bombay from the plague, caught while attending Dr. Manser through his last illness. Nurse Joyce was on the staff of the St. George's Hospital, Bombay.—July 5th is the date fixed for the grand "garden fête" in the grounds of the Chelsea Hospital in aid of the "Nurses for Soldiers and Sailors' Families."—Commander Blount, R.N., asks us to state that articles of every description are much needed for sale at the Victoria Hospital stall at the forthcoming Imperial Fête Bazaar, and will be gratefully acknowledged by him at the Victoria Hospital for Children, Queen's Road, Chelsea, any time up to Monday, June 14th.—District Nurses should all read the excellent advice which is being given in *Nursing Notes* under the heading "Incidental Opportunities of District Nursing." The articles are written with much practical good sense.—Miss Gane, who has just given up district nursing in Padiham to take the matronship of the Heath Memorial Convalescent Home, Llanfairfechan, was presented on leaving with a purse of money, in recognition of her "untiring labour amongst the sick poor."

### Pharmacy and Dispensing for Nurses.

By C. J. S. THOMPSON.

### VII.—IMPLEMENTS AND APPARATUS EMPLOYED IN DISPENSING.

THE dispensing counter or bench for compounding medicines should be furnished with a water tap and basin, gas arrangement for Bunsen burner, shelves above for bottles, jars, and measures, and drawers below the counter to hold other necessary requisites.

It will be well, perhaps, at this point to give a short description of the implements used in dispensing. To commence with, a good pair of apothecaries' scales, for weighing solids, are of the greatest importance to the dispenser. They must be perfectly accurate, and should turn to half a grain, care being taken to keep them scrupulously clean. The pans should be rubbed with a dry cloth each time after they have been used. Grain weights from half to six, and apothecaries' weights from half a scruple to four drachms, should be kept in a box close to the scales. In using the scales they should be held with the left hand, the pans being allowed just to touch the counter, in order to steady them. The weight must be placed in the left, and the article to be weighed in the right pan, a small quantity being put in at first, and then added to gradually. Between such addition raise the scales about an inch, in order to see if the correct balance has been reached, and add more, or reduce the quantity accordingly.

The pestle and mortar are the appliances used for powdering, pounding, and triturating medicinal agents. They are composed of metal, wedgewood or composition, and glass. The metal mortar is used for breaking up roots, barks, and other hard bodies, the wedgewood and glass being chiefly used for mixing or triturating purposes.

The measures employed in dispensing are of glass, and vary in capacity from one drachm to 20 ounces. The drachm measure is graduated in minims, the one and two ounce graduated in drachms, and the 10 and 20 ounce usually in ounces. When measuring a liquid the bottle containing it should be held in the right hand, the measure being in the left. The stopper of the bottle may be removed with the disengaged fingers of the left hand, and, on the measure being raised to the level of the eyes, gently pour the liquid into it until the desired graduated mark is reached. Carefulness in measuring is most important, and should never be hurriedly performed.

On removing a bottle from a shelf do not get into the habit of giving it a vigorous shake, or, when measuring, of pouring out two ounces instead of two drachms, and then having to pour the overplus back into the bottle.

Where drops are ordered the liquid should be dropped from the neck of the bottle. If it be a stoppered one, slightly loosen the stopper, and, supporting it against the inside of the neck with the forefinger, allow the liquid to escape drop by drop. The rim around the neck of the bottle should first be moistened. The drop is often confused with the minim. The former is by no means an accurate method of measuring a liquid, and is now happily falling out of use. The drop may vary in size according to the consistency of the liquid, the shape of the bottle from which it is dropped, and the amount of liquid in it at the time. Therefore it must be remembered that drops and minims are not synonymous terms. Funnels of varied size, constructed of earthenware or glass, &c., are used in pharmacy for filtering or straining. In dispensing, the smaller sizes of from two to four ounces in capacity are generally used. The filtering media usually employed is unsized paper known as "filtering paper," and may be bought in packets, ready cut, circular in form, or in sheets. The paper may be simply folded into the form of a cone or plaited concertina fashion, then placed in the funnel and the liquid carefully poured down the side of the filter to prevent the paper breaking. For straining a liquid, to remove any foreign substance or dirt that may have got into it, the medium used must depend upon its consistency. For instance, for thick syrupy fluids flannel or fine muslin may be used; for thin watery solutions absorbent wool or tow is best, it being simply necessary to place the medium in the neck of the funnel and run the liquid through it. When it is necessary to filter a large quantity of liquid containing heavy solid matter, it is a good plan to use two thicknesses of paper, or place a second small filter paper arranged as a cap to strengthen the cone and prevent breakage. The folded filter paper should be somewhat smaller than the funnel in which it is placed, as a projecting, badly cut, or torn filter, wastes the liquid both by absorption and evaporation. The paper itself should never be quite filled with liquid, but a margin of about an inch left at the top. When filtering liquids that rapidly evaporate on exposure to the air, such as alcohol, the top of the funnel should be covered over.

The pill machine is the appliance used for rolling and cutting a mass into pills. It consists of two parts, one of which rests on the counter, while the other is used with the hands. The latter consists of a flat piece of wood about three or four inches wide with a handle at each end. One side is used as a roller, while the other, to which a brass plate with hemispherical grooves running across in parallel lines, is attached, is employed as the cutter. The larger and stationary part of the machine also consists of two portions, one being flat for rolling, and the other fitted with a corresponding grooved brass plate which exactly coincides with those in the other part.

The operation of rolling and cutting a mass into pills is carried out in the following manner. The mass having been worked to a proper and plastic consistence, is rolled out on the flat part of the machine by pressure with the upper part into a pipe or long cylinder of the required length. It is then placed across the metal grooves, the cutter being placed over it, and then by slight pressure and a sharp rolling movement, the pipe is cut and formed into pills. The pills are rounded or finished by being placed under a perfectly smooth disc of hard wood, having a rim to prevent them escaping, and being rapidly revolved on the flat part of the pill board. The ordinary pill machines are made to cut from 12 to 24 pills of from 1 to 5 grains in weight.

The plaster spatula used for spreading plasters on leather or other base, consists of an oblong iron blade about four inches in length, which is attached to a curved shank having a wooden handle. The under edges of the blade are bevelled and the whole sometimes slightly curved. Another form of spatula is heated with gas. It is made hollow throughout and connected with the gas pipe by means of a rubber tube. The upper part of the blade is perforated with small holes, through which the gas passes, and on being ignited soon warms the metal and keeps it at a suitable heat. Suppository and pessary moulds are usually made of gun metal and nickel plated. They vary in size, and are constructed to make six, twelve, or twenty-four cones. They divide down the centre into two sections, which are held together by means of a screw.

Palette knives and spatulas for mixing ointments, &c., are made of metal, bone, and vulcanite, and may be had stiff or pliable.

A few small porcelain dishes, an iron tripod stand, glass stirring rods, glass flasks and beakers, all go to make up the practical furniture of the dispensing counter.

### Post-Graduate Clinics for Nurses.

By a Trained Nurse.

### IX.—TACT AND TEMPERAMENT.

In the last article some suggestions were offered as to practical methods of arranging the hours, meals, and recreations of each, when two nurses are engaged on a private case. Some mention may now be made of the personal relations of the two workers, for on the smoothness of these depends much of the success—social and professional—of their ministrations. Now, though this statement may sound somewhat paradoxical, a case in private practice may prove infinitely more trying when two nurses are engaged in the duty than when only one has full care and responsibility. For where two personalities are involved the possibility of friction and playing at cross-purposes immediately arises, especially if either happen to be of an "incompatible" temperament. Happily, however, in the large majority of instances nurses in private practice work cordially and cheerfully together; but, when this is the case, it is frequently because of the good-will and tact of one, and in rarer cases of both. When three nurses are gathered together in a sick-room—and this I have frequently had experience of in the United States—clouds are apt to darken and threaten, and resultant breaks and rifts can be avoided only by great tolerance and good humour. These last two qualities are the most valuable possessions in all human intercourse, and large drafts will be made on them when a case is put into the hands of three nurses.

"I invariably arrange," said a wise and experienced superintendent of a nursing institute in New York, "when two or more nurses are required at the same time on one case, that both or all shall be graduates of the same Training School. I find by practice that this precaution lessens the danger of friction. Consequently, instances of want of harmony between such nurses when engaged on the same case is rare indeed. On the other hand I have proved repeatedly that nurses from different Training Schools do not get on so well together, and that the position of affairs is apt to become uncomfortable. Every good nurse should be, and generally is, imbued with a strong wish to uphold the status and traditions of her Training School and Hospital. Indeed, I find this desire to leave a good impression of her school on the minds of her patient and his friends is a stronger check on a tendency to bad temper or superficiality in nursing method than any consideration of a more personal nature. To keep up the honour of her hospital is usually a stronger motive in the nurse than is a consideration of 'getting on in the world.' And it seems to me this is an admirable trait in a nurse's character, which we should do everything in our power to foster. Now it is quite reasonable to conclude that the presence and co-operation of another graduate of the same school will *increase* the desire of each one to add to the credit of the hospital from which she took her certificate. And, practically, I find it works out like that, so I make a rule, which I adhere to as rigidly as may be, in sending two nurses out to one case, that these two shall be pupils from the same training ground. So far I have put the case from the point of view of the personal harmony and cordiality which two nurses from the same hospital are likely to accord one another. But another aspect, which is of great importance to the patient, suggests that the methods and routine of nursing employed by each of such nurses would better fit in and accord with those of the other when each had been trained on the same lines. To illustrate exactly what I mean, we might take an instance of two tutors being required for one boy. If a graduate of Oxford and a graduate of Cambridge were selected, the education of the boy would surely suffer, since he would be for ever between two more or less rival systems of tuition, and the weakness of human nature would undoubtedly prompt each tutor to impress upon him the superiority of method attained by *his* 'Varsity. So with the two nurses. No two Training Schools are alike, and it is wilfully adding to the possible stumbling-blocks of the sick-room when we appoint two nurses from opposite schools to share the nursing of a sick person. Theoretically, good nursing is good nursing all the world over. But, practically, there are many ways—perhaps equally good—by which the same end may be attained. But, personally, I prefer that the merits and advantages of differing methods of poulticing, bandaging, and fomenting should not be argued out over the bed-side by two nurses who 'have been taught to do things very differently in their hospital.'" Of course, there is a certain amount of commonsense and truth embodied in these views, given as they were by a woman with some thirty years' experience in various departments of nursing. But, I think, if we were to take her statements quite literally, we should be guilty of an injustice to the rank and file of nurses, for, speaking generally, there is a freemasonry and *camaraderie* between nurses as a class —quite irrespective of and above the small rivalries and little jealousies unfortunately existing between some Training Schools. Some dispositions and hearts, however, remain narrow through a whole lifetime of contact with broadening influences, but there are other temperaments large and generous in spite of every disability. And the woman with the latter temperament was specially fitted and endowed by Nature for nursing work.

I always think — and I hope the statement will not lead to "faddiness"—that I can draw a very ready distinction between the nurse who reads the newspapers and the one who does not. Newspaper reading is not generally conceded to be an educational pursuit, and a fondness for the "daily" is supposed by some to argue an absence of intellectual capacity. But familiarity with the events and progress of the day, as chronicled in the daily press, has a widening and broadening effect on the mind which cannot be attained in any other way, and is far superior as a mental training to the reading of "many books." So when nurses consult me on little petty problems connected with something another nurse has done or said, or has not done or has not said, I frequently say, "My dear nurse, go and read the newspapers, think on the great events of the day, and the great people and interests of the world, and cease from troubling a narrowed little mind with trifles beneath the consideration of a grown-up woman." Nurse does not always like the administration of the tonic, but, like the continuance of many a disagreeable dose, "it is good for her in the end." To a private nurse the reading of newspapers is invaluable, since a knowledge of people and public things will make her a constant, good companion to both men and women patients, and will enable her to while away many an hour otherwise tedious to convalescents, besides being a source of interest and enjoyment to herself. For enabling one to forget the petty frictions of daily life commend me to the reading of a good, rousing Parliamentary debate! But, on the other hand, much as the nurse may personally enjoy the mental athletics indulged in at St. Stephen's, she must keep her political proclivities in a mysterious background. For the sick-room must be kept free from the slightest controversy. I remember once, when undertaking a private case, that of a man somewhat well known in the political world for a rigid Conservatism, his anxious father, in an interview beforehand, suggested in an agonised entreaty, "I do not, my dear young lady, attempt any stifling of your conscience, but I do sincerely trust you have no views on the political problems of the day. My son, I fear, would absolutely refuse the medicine were it offered by hands whose owner had the faintest taint of Radicalism!" I reassured him gravely, and the patient to this day labours under the impression that his nurse was more singularly free from "views" than any woman he ever met. This impression is the triumph and apotheosis of "his nurse!"

### The Metropolitan Nursing Association.

The twenty-first annual meeting of the Metropolitan Nursing Association was held at Grosvenor House on April 2nd, and was well attended. In the absence of the Duke of Westminster, Chairman of the Executive Committee, the chair was taken by Mr. W. S. Caine, vice-chairman. Archdeacon Sinclair. the Rev. A. L. B. Peile, the Rev. Dacre Craven, Dr. Arnold Brown, and Dr. Dixon were also on the platform.

The report, the adoption of which was moved by Mr. Caine, recounted "another year's steady progress in the great task of supplementing the work of the hospitals by nursing the poor in their own homes" Looking back not only to the birth of the association, but also to its coming of age, it was satisfactory to see the sound system and high standard which it inaugurated very generally recognised and adopted, and the work itself taken under the direct patronage of her Majesty. It was most necessary to maintain the home of the association upon an independent footing as the Central Training School for the supply of nurses primarily to the Queen's Institute, and one in which the highest standard of district nursing should be upheld; the committee, therefore, pleaded for continued and increased support. Mr. Caine, after alluding to the great services rendered to the association by Miss Gray, the superintendent, and by Mr. Dacre Craven, the hon. secretary, drew attention to one or two matters mentioned in the report. Seventeen probationers completed their training during the year. Of these, 15 were trained for the Queen's Jubilee Institute (with which the association was affiliated), one for the Rural District Nursing Association, and one was a lady from the United States, who came to train at her own expense with a view to nursing the sick poor in their own homes in Baltimore. Lectures, provided at the expense of the Queen's Jubilee Institute, had been given at the Central Home on "Hygiene," "Fevers," and "Diseases of Women," and recently a course of demonstrations in "Sick and Invalid Cookery" had been substituted for the "Fever" lectures. Mr. Caine drew attention to the fact that there was a difficulty in getting a sufficient number of probationers to train.

The Rev. A. L. B. Peile, who seconded the adoption of the report, spoke of the bond which existed between the Queen's Jubilee Institute and the Metropolitan Nursing Association, and of the great increase in the demand for trained nurses throughout the country. It was an amazing work that the nurses did, and the country was indebted to them for their generosity, love, and helpfulness. He considered that they ought to be immeasurably better paid than they were at present, so that they might be able to put by against a rainy day, and he trusted that the money which was this year being placed in the hands of the Institute might make possible an increase in the salaries of Queen's nurses. Mr. Peile alluded to the vexed question of "rural" nursing, and said he hoped Queen's nurses would not think that they were in any way disparaged by the recognition of the "rural" nurse on the part of the Institute. No one could become a "Queen's nurse" unless she were fully trained. The recognition of another class of nurse was only an attempt to make the best arrangement possible in rural districts.

A resolution was moved by Archdeacon Sinclair pledging the meeting to support the association, and speeches followed from Dr. Oswald Browne, Dr. Dixon, and Mr. Mocatta. The meeting concluded with the usual votes of thanks.

Miss Gray afterwards entertained a number of guests at tea at the Central Home, 17, Bloomsbury Square.

### Novelties for Nurses.

### SPRING DRESS MATERIALS.

"In the spring," to parody the words of a great poet, "a woman's fancy lightly turns to thoughts of dress." Varied and abundant are the novelties that are temptingly displayed for her choice, and few, if any, are more attractive than those produced by Messrs. Egerton Burnett, of Wellington, Somerset. This high-class firm has always been in the front rank in regard to serges, the quality and durability of which experience has placed beyond question. This year, in addition to a splendid selection of their celebrated "Royal serge," is included a new material which is likely to become extremely popular, viz., "Britannia serge coating." This warm and serviceable cloth is sold at 2s. 11d. a yard only, and is guaranteed everlasting wear. Among the samples are some charming homespuns, all wool, and unshrinkable. The white serges are very dainty and would make ideal boating costumes. On the Riviera they are all the rage this season, and every well-dressed woman includes a white serge costume in her wardrobe. An estamene coating in red is very fascinating, and cannot be called dear at 1s. 9½d. per yard, double width. There are some pretty greys, both light and dark, that rather appeal to the quiet dresser, and would make up very stylishly. Nurses, however, will be chiefly interested in the large assortment of cloakings, most of which are sixty inches wide, and splendid value for the money. These can be used equally well for costumes, and being thoroughly waterproof, would be invaluable for country wear. District nurses, who often have long distances to traverse in all conditions of weather, could not do better than to invest in a cloak or dress of this admirable material. There is an excellent selection of washing materials which will charm the most fastidious. Zephyrs in delicate blue and white check are found varied with strong twills in darker blue, relieved by narrow white stripes. A grey camelet is most bewitching, and would make a useful, and at the same time most becoming holiday suit. The brown hollands are always cool and clean-looking, and the "Nightingale" stripe in green, black, and blue is a novelty that promises to become popular. White linen for aprons, forty inches wide, and of good quality, we notice is quoted at 1s. 7½d. per yard. The tailoring department, which has only been established three years, is a great convenience to customers who want a really well-made costume at moderate price. By a simple system of self-measurement, particulars of which are furnished post free on application, an accurate fit can be obtained without the trouble of a personal interview. Gentlemen as well as ladies can be fitted, and in a most satisfactory manner, as the increasing number of clients agreeably testifies. Egerton Burnett and Co. do not, however, limit their business only to what we have been describing. The underclothing department is a very extensive one, and includes hosiery, vests, combinations in silk and wool, which are ideal wear for warm climates. Pure cachemire wool nightdresses are a speciality that will be irresistible to the rheumatic, for not only are they warm and light, but they are beautifully finished off with lace trimmings. Rugs and blankets are found in great variety, and the popular fancy striped Austrian blankets can be purchased at 6s. 6d. each. Hold-alls, travelling bags, and trunks complete the list, and maintain the high quality that is the distinguishing mark of all goods sent out by this enterprising firm.

### Where to Go.

Victoria Commemoration Club.—Dr. Ewen J. Maclean, M.D. Edin., M.R.C.P. Lon., delivered the second lecture in the course on gynæcology he is giving at the Victoria Commemoration Club, 29, Southampton Street, Strand. Messrs. Maw, Son, and Thompson lent a complete set of instruments, which Dr. Maclean thoroughly explained. The next lecture is on Tuesday, the 13th inst., at three p.m., when the subject will be "Clinical Signs and Symptoms of Special Conditions, Abortion, Vaginal Discharges, &c."—Miss Earls' demonstration was, as usual, delightful. *Omelette souflé*, invalid soup, barley water, grilled chops, and fried fish were as dainty and appetising as heart could wish.

### A Case for Government Action.

INGS' HOUSE NURSES CO-OPERATION.

For some years London has been free, with slight exceptions, from any attempt on the part of interested persons to promote so-called hospitals. We have long been aware of the efforts made in all directions to procure money for the purpose of furthering schemes promoted by the so-called Ings' House Nursing Co-operation. The latest of these schemes is described in the *Morning Post* of the 23rd ult. as a new hospital for the reception and treatment of paying patients situated in Oxford Street, near Oxford Circus. This institution was opened by Lady Harris, who was accompanied by Lord Harris, and the company included Mr. Bhownaggree, M.P. We commend to Lord and Lady Harris' careful consideration the following account, which appeared in the daily papers, of proceedings at Lambeth County Court on March 31st, nine days later, which give an outline of the treatment to which patients have been subjected at the Ings' House, which is under the same management as this so-called hospital:—

"Judge Emden, sitting at Lambeth County Court yesterday, concluded the hearing of the action of Canning v. Sivell. It was a claim by Miss Margaret Canning, the proprietress of the Ings' House Nursing Co-operation, 81, New Bond Street, W., against Mr. Sivell, a retired gentleman, living at Croxted Road, Dulwich, for the balance of an account of £34 13s. 6d. for board and lodging and other expenses incurred in connection with the nursing of Miss Sivell, defendant's daughter. Mr. A. B. Shaw was counsel for the plaintiff, while the defendant was represented by Mr. Lithiby, barrister. From the opening statement of Mr. Shaw it appeared that Miss Sivell was a victim of acute melancholia, and as the result of an interview between the plaintiff and Mrs. Sivell it was arranged that the young lady should be admitted to plaintiff's establishment. Mrs. Sivell expressed a wish that her daughter should be treated by the Weir-Mitchell process, which, said counsel, was a method combining electrical treatment with massage and special dieting. The plaintiff's case was that on her pointing out to Mrs. Sivell the very expensive nature of this process the latter consented that her daughter should undergo a process of massage treatment, together with a special dietary, at a charge of six guineas per week. Miss Sivell was admitted a patient on December 11th, and her case was under the immediate supervision of Dr. Forbes Winslow, the specialist on mental diseases. After she had been at the establishment a few weeks Miss Sivell was discovered to be suffering from diphtheric affection of the throat, for which she was attended by Dr. Beare. In connection with this there was a claim against the defendant for disinfection of the house and also for the supply of stimulants and special nursing. The plaintiff, who stated that she was a member of the London Obstetric Society, gave evidence in support of her counsel's opening statement. She managed a second establishment at 180, Oxford Street, which was publicly opened by Lord and Lady Harris last Monday week. Mrs. Robinson, who said she had had no experience in sick nursing, deposed to having been engaged by plaintiff to sit up by the young lady in December; and Nurse Clarke, who was formerly connected with plaintiff's "house," described the special dietary given to the patient, which, she said, consisted mostly of salt beef, white herrings, eggs bought at sixteen a shilling, and milk made from the cheapest brand of condensed tins. Dr. Forbes Winslow stated that one of the most important features of the Weir-Mitchell treatment was that the patient should receive a nourishing diet. He did not consider the food described as at all proper for a patient in the condition of Miss Sivell to have. Mr. Thomas Robinson, formerly secretary to Miss Canning, said the food for the patient was sent up from the dinner table. On one occasion Miss Sivell expressed a wish to have some fish. Thereupon Miss Canning gave him sixpence, with instructions that he should buy a twopenny piece of fish from a fried fish shop in Little Marylebone Lane. When he returned this fish was sent up to Miss Sivell with a potato "in its jacket." He certainly knew of no extra nursing having been provided for Miss Sivell.

Judge Emden said the action was of grave importance, having regard to the serious allegations that had been made as to the management of the place called the "Ings' House Nursing Co-operation." It was unnecessary for him to say that institutions of this description, which professed to deal with the mentally afflicted, should be very carefully managed. The case revealed the existence of a nursing venture—he could use no better word—in the very centre of civilisation, a venture which, although described as co-operative, was not co-operative except for the purpose of decoying the public. With regard to the terms of the contract between the plaintiff and Mrs. Sivell he had no hesitation, on the evidence and on the sequence of events, in holding that it was that the Weir-Mitchell treatment which should have been applied to the case of Miss Sivells. As to whether the contract was carried out, he had had the advantage of the evidence of a leading expert, Dr. Forbes Winslow, and taking the whole of the evidence in connection with the doctor's statement he was bound to come to the conclusion that the whole of the transactions on the part of the plaintiff were a deception; that the agreement was not carried out; that, further, she had neither the nurses nor the appliances to carry it out. Plaintiff's claim for spirits supplied to Miss Sivell was, having regard to the fact that the lady was mentally afflicted, most outrageous, not only in the light of evidence, but in the light of common sense. Plaintiff could not, under the circumstances, recover, and as to the counter claim he held that the improper treatment to which Miss Sivell was subjected entitled defendant to the return of the £19 5s. 9d. already paid to Miss Canning, and he said that he felt it his duty to award Mr. Sivell £5 as damages against the plaintiff. He should also give defendant costs on the higher scale."

Nurses should especially note that, in the course of her evidence, Miss Canning stated that "she had allowed over fifteen nurses to live in her house, and they worked among the patients for their board and lodging."

With unconscious irony the *Morning Post* heads its notice of the opening of the new establishment under the management of Miss Canning as "A New Hospital for Paying Patients." On Ings' House and its proceedings we do not wish at present to make any further comment; but this new so-called Hospital for Paying Patients has been opened in a public manner under the patronage of well-known names, and has appealed for monetary support from the public. Under these circumstances it is but right that the fullest publicity should be given to the above facts in regard to those by whom it is managed. We venture to hope that Lord and Lady Harris and Mr. Bhownaggree will at once withdraw their names, and that the latter will put a question to the President of the Local Government, asking whether it is not possible for steps to be taken to vest County Councils or the Government with sufficient authority to protect the lives and persons of her Majesty's lieges from such "adventure hospitals," and the philanthropic public from the discredit and loss of subscribing to them under the impression that they fulfil some public purpose. Anyone who receives an appeal from Miss Canning for funds should, before replying, communicate with Mr. C. S. Loch, secretary of the Charity Organisation Society, 15, Buckingham Street, Adelphi, W.C.

### Everybody's Opinion.

[Correspondence on all subjects is invited, but we cannot in any way be responsible for the opinions expressed by our correspondents. No communication can be entertained if the name and address of the correspondent is not given, or unless one side of the paper only is written on.]

### ALMSHOUSES FOR NURSES.

Nurse E. writes: It was with great pleasure I read in last week's Hospital the kind letter from "A Trained Nurse" suggesting a home or almshouses for nurses in ill-health or too old for work. A home would be a boon! It does seem strange that no provision of this kind has been made for nurses broken down in health. I have known of some sad cases without homes or means. At St. Leonards there is a home for ladies of "limited means" (Albert House), which offers two furnished rooms, cooking, and attendance, 8s. per week. Could not some such home for nurses be provided in Bournemouth? If at 7s. per week so much the better. The mild climate (invaluable to many) and lovely scenery are especial recommendations, and its being such a health resort for invalids it brings a large number of nurses into the town. I, indeed, should be most thankful for such a home, from ill-health. I am unable to work, and for the last two years have experienced the discomforts and loneliness of lodgings with "limited means."

### A "UNIFORM" QUESTION.

"Bootle" writes: Regarding the "uniform" question recently touched upon by "A District Nurse" as to the advisability of the wearing or not wearing of uniform for evening wear, I would venture to suggest that uniform should not be worn when invited out for the evening. The nurse invited is supposed to meet the other guests on a social footing, and not in the capacity of a nurse. Therefore, with that in view, the uniform would be out of place, and most decidedly not in keeping with a social gathering. "The alpaca or navy blue silk dress, made plainly," would serve the purpose well. But why not have some soft lace instead of the uniform collar and cuffs? I am of opinion that both indoor and outdoor uniform should be rigorously excluded from theatres, concerts, socials, &c.; and that nurses should upon those occasions wear their private clothes. If this custom became universal, what a boon it would be to the profession *en masse*. We should then know that the silly giggling creatures whom we sometimes meet in theatres are not nurses, but shams, attired so merely to attract attention.

"A. M. F. C." writes: In reply to a "Uniform" question I have found that for a district nurse it is most convenient and economical to have only uniform and evening dress. Why is a fancy uniform in silk less expensive than an ordinary dinner gown in the same material? Nothing needs a more first-rate making than the severely simple style, whereas an ordinary dressmaker can make an evening dress. I find my working gowns, a well-made stuff uniform gown that does for anything before dinner, a few simple evening blouses suitable for quiet dinners with my friends, and two better ones good enough for swell occasions, the fittest and cheapest style of dressing. I always wear uniform out of doors, for it is gloves and boots and changing fashions in hats and jackets that make dress run up. And on the few occasions when a sudden call might be feared during an evening out, I should strap up my cotton gown, &c., and take it with me. There is a sort of affectation, to my mind, in imitation uniform, and a discomfort in substantial stuff dresses for evening wear. As we must get a few decent dresses for our holidays, why not dress like other people in those same gowns during evenings spent in civilisation? After all there is a good deal of licence in fashion nowadays, and seldom as we district nurses get out of uniform we can wear out a dinner gown in a few years, holiday wear included.

### THE PLAISTOW MATERNITY CHARITY.

We are asked to find space for the following appeal:—

Sir,—Will you allow us through the medium of your columns to plead the cause of a humble charity which, in this year of overpressure, stands a serious chance of being forgotten. We mean Sister Katherine's admirable nursing work among the poor of Plaistow and West Ham. As your readers know, there are very few well-to-do people in that part of London. Dock labourers, factory operatives, and the helpless drifting population without regular employment, make up the vast mass of the inhabitants. There are scarcely any general hospitals, and the provision for those who are unfortunate enough to be hurt or seized by disease, is wholly inadequate to the needs of the district. These nurses also do a splendid work among the poor mothers of the parishes in which they work (fourteen in number). All who know anything of the London poor must have been struck by the extreme discomfort, and frequently unnecessary danger to which women are exposed at the time of their confinements by the entire absence of anything which can be called nursing. Good nursing is necessary at such times under the most favourable circumstances, but how much more when four or five other people share the miserable tenement? Finally we would plead with country people to give a helping hand to this charity, the great training school for village nurses. East and west, south and north, the Plaistow nurses have gone, carrying the benefits of their teaching to many a poor mother and sick man. County councils from all parts of England have sent pupils to be trained there, besides numberless private associations. We would earnestly beg the public not to let such a good work cease for want of a little timely help.—We are, &c.,

Maud Selborne,
E. Ebury
(Members of the House Committee).

Subscriptions or donations may be sent to Robert Williams, Esq., treasurer of the Maternity Charity and District Nurses' Home, Howard's Road, Plaistow, E., or direct to the bankers, Messrs. Williams, Deacon, and Co., 20, Birchin Lane, E.C.

### Appointments.

H.R.H. Princess Christian's Trained Nurses, Windsor.—Miss Mary Robin has been appointed Superintendent of this home in place of Miss Simpson, whose resignation we notice elsewhere. Miss Robin was trained at the Royal Hospital for Sick Children, Edinburgh, at the Edinburgh Royal Infirmary, and the Glasgow Maternity Hospital (L.O.S. certificate). She is a Queen's nurse (Scottish branch), and has held the position of superintendent of the District Nurses' Home, Kilmarnock, and that of assistant and district superintendent at the Training Home for Queen's Nurses, Edinburgh. Recently Miss Robin has held the appointment of assistant superintendent at the Royal Infirmary, Edinburgh, in charge of the Nurses' Home.

Private Nursing Institution, Grafton House, Bolton.—Miss Mari Clark has been appointed Lady Superintendent of this institution. Miss Clark (known as "Sister Mari") holds a three years' certificate of training from the Liverpool Northern and Bath Royal United Hospitals. On completing her training, she acted as sister and assistant matron at the Bath Royal United Hospital, and subsequently filled the post of night superintendent and deputy-matron at the Bolton Infirmary and dispensary. During the last five years Miss Clark has been matron of the Bolton Borough Hospital. She holds excellent testimonials.

Glasgow Maternity Hospital.—Miss J. A. Husbant has been appointed Matron of this hospital. Miss Husbant holds certificates from the Royal Infirmary, Edinburgh, the Royal Maternity, Edinburgh, and the London Obstetrical Society. For the past five years she has been charge nurse and practically assistant matron at the Royal

Maternity Hospital, Edinburgh, and now leaves accompanied by the best wishes of medical and nursing staffs for her success in her new sphere of work.

The Heath Memorial Convalescent Home, Llanfairfechan, N. Wales.—Miss Gane has been appointed Matron of this home. She was trained and certificated at Chestes Infirmary, and for the last five years of her stay there war sister-in-charge of the principal men's wards. Miss Gane afterwards joined the Staffordshire Nurs'ng Home, and took up district nursing in Padiham, where she remained nearly six years.

### Minor Appointments.

Sheffield General Infirmary.—Miss Elizabeth Duffield has been appointed Sister of the Women's Surgical Wards at this institution. Miss Duffield was trained at the Royal Hospital, Belfast, and afterwards gained experience in private nursing in connection with the Belfast Nurses' Home and Training School. She was subsequently appointed night charge nurse in the men's and women's surgical wards of the Royal Hospital, leaving to take charge of the women's medical and surgical wards at the County Infirmary, Downpatrick, where she has been for the past year.

Sea Bathing Infirmary, Scarborough.—Miss Beatrice de Montmorenci and Miss Helen Oliver have been appointed Charge Nurses at this infirmary. Miss de Montmorenci trained for three years at Bristol Royal Infirmary, and also holds the L.O.S. diploma. Miss Oliver was trained at the Clinical Hospital, Manchester, where she worked altogether for five and a half years, latterly as ward sister. She has also had experience in district nursing for the North London Nursing Association. Both have excellent testimonials.

Government Hospital, Accra.—Miss Alice M. M. Williams has been appointed Nurse at this hospital, and sails this week for her new post. Miss Williams was trained at Charing Cross Hospital, afterwards working as district nurse for a year at Woking, and subsequently joining the Nurses' Co-operation, 8, New Cavendish Street. Recently she has been private nursing in Switzerland and Russia, and has only just returned to England.

Cardiff Union Children's Hospital at Ely Schools.—Miss Kate C. Ryan has been appointed Nurse at this institution. She received her training at the Queen's Hospital, Birmingham, and has since worked as assistant nurse at Stockport Union Workhouse, as nurse at the Blue Coat Hospital, Liverpool, and night nurse at the Midland Eye Hospital, Birmingham. Miss Ryan has also had experience in district nursing.

Beckett Hospital, Barnsley.—Miss J. C. Scully has been appointed Charge-Nurse at this hospital. Miss Scully was trained at the North Riding Infirmary, Middlesburgh, remaining there for four years as charge-nurse. Since then she has had experience as charge-nurse for two years at Darlington Hospital, at Dr. Christopher Martin's Private Hospital for Women, and at Hartlepool Hospital.

Derbyshire Royal Infirmary.—Miss Edith A. Cowley has been appointed Sister at this hospital. Miss Cowley was trained at St. Bartholomew's Hospital, where she afterwards held appointment as staff nurse. Subsequently she was appointed sister of men's landing at the Salop Infirmary, where she has remained for 2½ years.

General Hospital, Great Yarmouth.—Miss Anna Elizabeth Edwards has been appointed Night Sister at this hospital. Miss Edwards received her training at the Hereford General Infirmary and has had experience in private nursing in connection with that hospital.

### Notes and Queries.

*Dispensing.*

(199) Should one learn more Latin than is used technically in dispensing when preparing for a pharmaceutical exam.? Also what is the price of the British Pharmacopœia, and where can I get it?—*F. B.*

You will find a list of books recommended in an article on "How to Become a Dispenser" in this week's "Mirror." The "British Pharmacopœia" is published by Spottiswoode for the General Medical Council, price 6s.

(200) What book would you recommend to a lady learning dispensing which would help her with Latin terms? Please also recommend a helpful book on midwifery for an L.O.S. candidate?—*Pamela.*

(1) Read the article on "How to Become a Dispenser" in *The Hospital* for April 3rd. (2) Herman's "First Lines in Midwifery"; Haultain's "Practical Handbook of Midwifery." Both books can be obtained through the Scientific Press, 28 and 29, Southampton Street, Strand, London, W.C.

*Advice Wanted.*

(201) Please tell me (1) if as good training is to be had in a cottage hospital as in a general hospital; (2) what hospitals or infirmaries wear strings to the caps, and can they be worn or not as one likes?—*C. de V.*

There is very good training to be had in some cottage hospitals, but if you intend taking up nursing this should only be as a preliminary to further training in a general hospital of recognised standing. Read "How to Become a Nurse" (Scientific Press, 28 and 29, Southampton Street, Strand, W.C.) (2) You will find sketches of the caps worn at most of the London hospitals in the above-mentioned book. Of course, if you enter a hospital you have to wear whatever the uniform at that particular institution may be.

*Training.*

(202) I wish to become a nurse. Please tell me what books to read. I am nearly eighteen.—*M. Roberts.*

You are much too young to begin nursing yet awhile. Read the book recommended above, "How to Become a Nurse," and till you are old enough to enter a hospital you can be learning something of elementary anatomy and physiology, a knowledge of which will be helpful to you later.

*Nurses' Co-operations.*

(203) Please tell me how to register a nurses' co-operation, and give me any information in your power about starting one?—*E. M. P.*

The most successful and best managed institution of the kind is the Nurses' Co-operation, 8, New Cavendish Street, W. Doubtless if you wrote to Miss Philippa Hicks, the lady superintendent, and asked her advice, she would be pleased to help you, and would send you the rules upon which that association is founded for your guidance. You seem, from your letter, to be setting about your scheme in quite the right way.

*Training.*

(204) Please tell me if any hospitals take probationers of 18 years of age, and what is the usual period of probation?—*L. H. R.*

All letters requiring an answer in this column must be accompanied by full name and address for the Editor's information (not necessarily for publication).

*Cottage Hospitals.*

(205) Where can I find out all about cottage hospitals, from their origin and construction to details of management, &c.—*Sister Edith.*

Burdett's "Cottage Hospitals," published by the Scientific Press, 28 and 29, Southampton Street, Strand, W.C., will tell you all you want to know about cottage hospitals.

*Training and Health.*

(206) "*M. D.*" writes about her health, and asks if she would be considered suitable to enter a hospital as probationer.

We should advise "M. D." to consult a doctor in the first place, and if he thinks her strong enough for nursing to make application at the hospitals.

*Midwifery Training.*

(207) I have been (after hospital training) working for some years as a private nurse. Would you advise me to expend some of my savings in taking a course of midwifery training, and if I did so, should I be likely to get plenty of work, and how should I get cases to start with? What would be the fees, and how should I set about obtaining the necessary training?—*Nurse Lena.*

Of course a nurse adds greatly to her marketable value by training in midwifery, and if she has the necessary qualifications is generally sure of work. At the lying-in hospitals a register is kept, on which midwives and nurses who satisfactorily complete their training can enter their names for a fee of 5s. We should advise you to write for advice to the Secretary of the Midwives' Club, 12, Buckingham Street, Strand, W.C., enclosing a stamped envelope for reply.

*Training.*

(208) Please tell me if a nurse trained at one of the London infirmaries would be received at the London or Guy's Hospital, or would she be obliged to enter as a probationer, and have a second course of training?—*Eltham.*

The large hospitals, as a rule, only promote their own nurses to be charge nurses or sisters, and we doubt if either hospital would accept as probationer an already trained nurse. You had better write yourself and ask the question of the matrons of the hospitals in question.

*Nurses' Hostels.*

(209) Can you tell me of any homes or "hostels" where a private nurse could reside on reasonable terms when disengaged?—*Nurse B.*

Write to Miss C. J. Wood, Nurses' Hostel, 27, Percy Street, Tottenham Court Road, W.C.

